# A natural inhibitor of kidney-type glutaminase: a withanolide from *Physalis pubescens* with potent anti-tumor activity

**DOI:** 10.18632/oncotarget.23058

**Published:** 2017-12-08

**Authors:** Canrong Wu, Mengzhu Zheng, Suyu Gao, Shanshan Luan, Li Cheng, Liqing Wang, Jiachen Li, Lixia Chen, Hua Li

**Affiliations:** ^1^ Hubei Key Laboratory of Natural Medicinal Chemistry and Resource Evaluation, School of Pharmacy, Tongji Medical College, Huazhong University of Science and Technology, Wuhan 430030, P.R. China; ^2^ Wuya College of Innovation, School of Traditional Chinese Materia Medica, Key Laboratory of Structure-Based Drug Design and Discovery, Ministry of Education, Shenyang Pharmaceutical University, Shenyang 110016, P.R. China

**Keywords:** KGA inhibitor, withanolide, physapubescin K, synergistic effects, structure-based virtual ligand screening

## Abstract

Kidney-type glutaminase (KGA), a mitochondrial enzyme converting glutamine to glutamate for energy supply, was over-expressed in many cancers and had been regarded as a promising therapeutic target in recent years. Structure-based virtual ligand screening predicted physapubescin K, a new withanolide from *Physalis pubescens*, to be potential KGA inhibitor. Enzyme activity inhibition assays and microscale thermophoresis experiments had demonstrated the efficiency and specificity of physapubescin K targeting KGA. Additionally, physapubescin K exhibited potent proliferation inhibitory effects on a panel of human cancer cell lines, such as SW1990 and HCC827-ER. It blocked glutamine metabolism in SW1990 with increasing intracellular level of glutamine and decreasing glutamate and its downstream metabolites. Physapubescin K also significantly inhibited the tumor growth in a SW1990 xenograft mouse model. Interestingly, physapubescin K could reverse the resistance of HCC827-ER cells to erlotinib and synergize with the hexokinase 2 inhibitor to markedly enhance the inhibition of SW1990 cell proliferation.

## INTRODUCTION

Since Otto Warburg’s pioneering work on aerobic glycolysis [1] was published, glucose has been a focus for cancer metabolic research. In recent years, researchers gradually found that other nutrients such as glutamine also played important roles in cancer metabolism [2–4]. Glutamine addiction, like Warburg effect, was also associated with dysregulation of metabolism pathways involved in cancers. As glucose was rapidly absorbed and converted to lactic acid, tumor cells were proposed to increase their use and uptake of glutamine, thereby producing alpha-ketoglutaric acid as the complementally fuel in Krebs cycle, as well as intermediates for the synthesis of lipids, nucleosides and other biomolecules required for cells’ proliferation and survival [5, 6]. Owing to the addiction of many cancer cells to glutamine, blocking glutamine metabolism was considered to be an attractive strategy for cancer therapy.

Gutaminases are mitochondrial enzymes that control the first step in the glutaminolysis pathway by converting glutamine to glutamate. Glutaminases possessed multiple tissue-specific forms, and was encoded by two genes in mammals. Kidney-type glutaminase (KGA) and glutaminase C (GAC) were encoded by GLS1 and liver-type glutaminase (LGA) was encoded by GLS2 [7] GLS1 was broadly expressed in normal tissue and over-expressed in many cancer cells, thus played an important role in tumor metabolism [8]. Whereas GLS2 expression was restricted to the liver, brain, pancreas and pituitary gland. The dependence of cancer cells on glutamine metabolism made GLS1 an attractive anticancer target. It was reported that some oncogenes including c-Myc, Raf, Ras and Rho GTPase could up-regulate KGA expression in many cancer cells [7, 9, 10, 11, 12]. Treating tumor cells with KGA specific siRNA induced apoptosis under oxidative stress [13]. Therefore, KGA is proved to be an emerging target for cancer therapeutics [7, 12, 14, 15]. Although the potential benefits of KGA inhibition have been recognized for nearly 10 years, small molecule KGA inhibitors were relatively scarce, and merely few chemically synthesized derivatives, such as DON, BPTES and CB-839 were available until now [7, 14]. Among them, only CB-839 has moved on to clinic phase I trials [6, 16, 17]. Nevertheless, some shortcomings like non-specificity, low solubility and moderate potency may limit its pharmacological applications.

Natural products have been acknowledged to be an important source for antitumor drug discovery and development [18]. Our group is making efforts to find small molecule KGA inhibitors with high potency and selectivity from natural products [19]. About 500 compounds from a small in-house database of natural products were screened against the KGA model *in silico*, as a result, a new withanolide with characteristic δ-lactol side chain, physapubescin K from *Physalis pubescens* L., was discovered as a new natural KGA inhibitor, which showed potent anticancer activity both *in vitro* and *in vivo* and displayed synergistical inhibition of tumor cell growth with benserazide and erlotinib.

## RESULTS

### Structure elucidation

**Figure 1:**
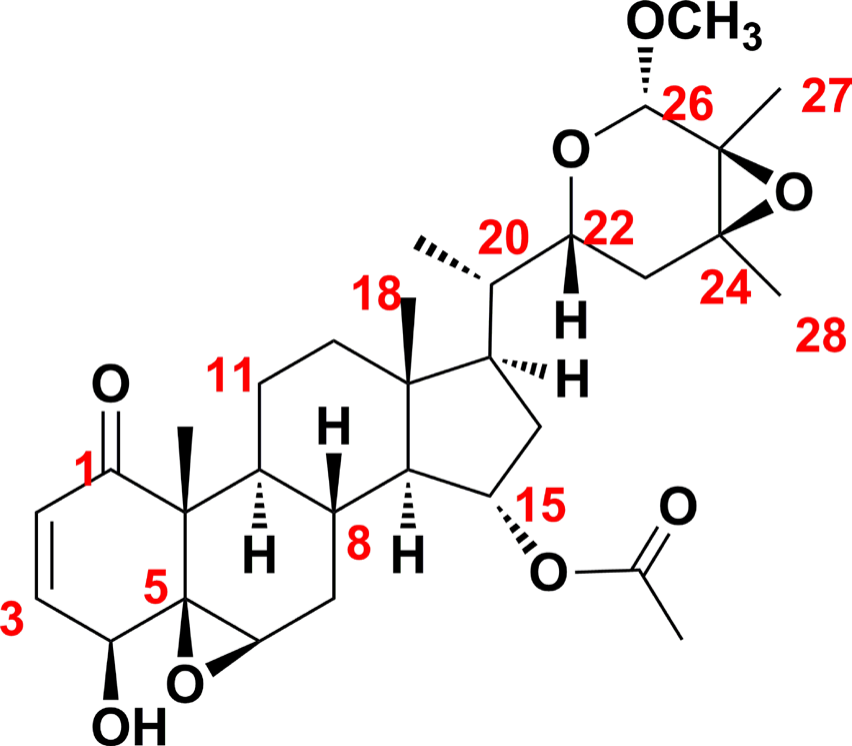
Chemical structure of physapubescin K

The molecular formula of physapubescin K was determined to be C_31_H_44_O_8_ via HRESIMS and NMR data. The NMR spectrosopic data (Table 1) suggested that physapubescin K possessed the typical withanolide skeleton, and it closely resembled physapubescin [20] except for an additional methoxyl signal [δ_H_ 3.41 (3H, s), δ_C_ 56.4]. In the HMBC spectrum, the signal at δ_H_ 3.41 correlated with δ_C_ 101.4 (C-26), and δ_H_ 4.50 (H-26) correlated with the methoxy carbon at δ_C_ 56.4, indicating that the methoxyl was attached to C-26. The NOESY correlations between H-22 and H-26/H-23_eq_, between CH_3_-27 and CH_3_O-26, and between CH_3_-28 and H-23_ax_, suggested that the CH_3_O-26, CH_3_-27, and CH_3_-28 were *trans* to H-22. The 20*S*,22*R*-configuration of physapubescin K was determined through the characteristic coupling pattern of H-22: δ_H_ 3.47 (1H, dt, *J* = 11.5, 2.9 Hz) and NOESY correlations of H_3_-21/H-23_ax_, H-17/H-23_eq_, H-16α/H-22, H-16β/H-22, and H-22/H-20 [21, 22]. Therefore, the structure of physapubescin K was assigned as shown in Figure 1.

**Table 1:** ^1^H NMR (600 MHz) and ^13^C NMR (150 MHz) spectroscopic data of physapubescin K in CDCl_3_, δ in ppm

position	Physapubescin K^a^	
	δ_H,_ (J in Hz)	δ_C_
1	−	202.1
2	6.13, d (10.0)	132
3	6.89, dd (10.0, 5.9)	142.3
4	3.69, d (5.9)	69.6
5	−	63.5
6	3.13, brs	61.8
7	2.04, dd (15.0, 4.1)	30.2
	1.42, m	
8	1.64, m	29
9	0.97, td (11.4, 4.1)	43.7
10	−	47.5
11	1.76, m	21.7
	1.39, m	
12	1.86, dt (12.5, 3.5)	39.3
	1.13, m	
13	−	42.9
14	1.19, m	58.7
15	4.77, td (9.4, 2.7)	76
16	2.03, m	37.2
	1.46, m	
17	1.23, m	49.9
18	0.68, s	12.6
19	1.34, s	17.3
20	1.61, m	38.8
21	0.83, d (6.5)	12.7
22	3.47, dt (11.5, 3.0)	69.4
23	1.73, dd (14.4, 11.5)	29.6
	1.59, dd (14.4, 3.0)	
24	−	62.6
25	−	61.3
26	4.50, s	101.4
27	1.24, s	14.6
28	1.28, s	19
AcO-15	1.96, s	170.7
		21.3
OMe	3.41, s	56.4

### Virtual ligand screening

**Figure 2:**
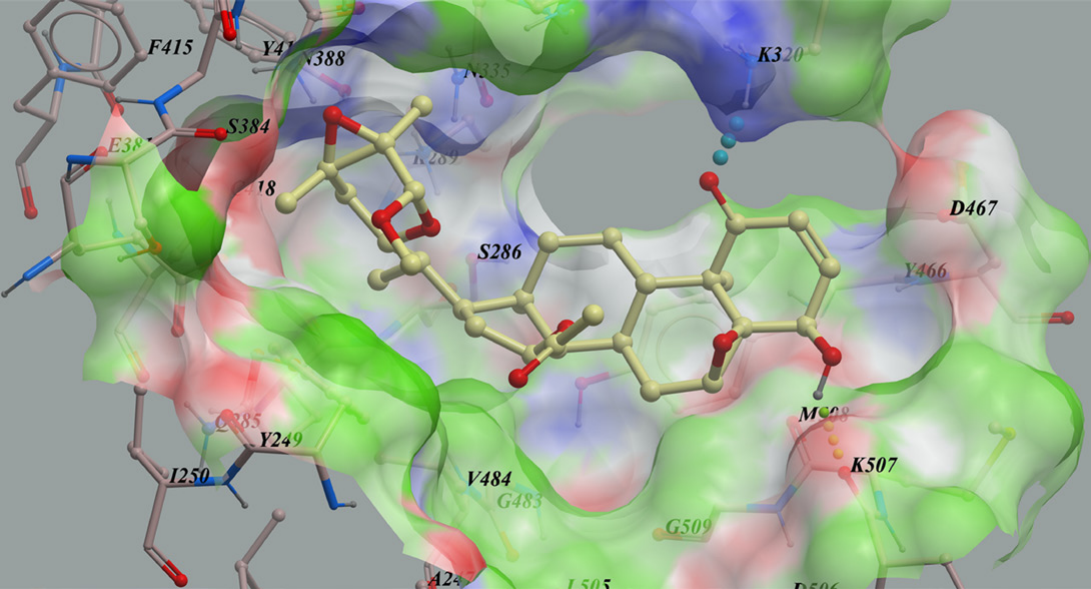
The low-energy binding conformations of physapubescin K bound to KGA generated by virtual ligand docking

To find new KGA inhibitors, 500 compounds from the in-house natural product library were screened against the KGA model *in silico* based on its X-ray structure (PDB code: 3VP1) [23]. Compounds with lower calculated binding energies were considered to have higher binding affinities with the target. The results predicted that physapubescin K exhibited the highest binding affinity to KGA with the most negative mfScores of -159.12 (Table 2). From the generated docking model, physapubescin K was adopted an extended conformation, which occupied the active site of the enzyme, hydrogen bonds were predicted between ketone group at C-1 and Lys320, as well as between hydroxyl at C-4 and carbonyl of Lys507. Also, physapubescin K formed key hydrophobic interactions between δ-lactol ring and Tyr249, and between ring D and V484. The π- π stacking interaction formed by ring B and Tyr466 further strengthens the binding (Figure 2).

**Table 2:** Binding affinity and inhibitory activity of physapubescin K (PSBK) against KGA enzyme *in vitro*

No.^a^	mfScores(kcal/mol)^b^	KGA	
		Kd (μM)^c^	IC_50_ (μM)
PSBK	−159.12	3.03 ± 0.38	0.52 ± 0.06
CB-839	N/A	0.81 ± 0.11	0.97 ± 0.04
BPTES	N/A	u.p	8.37 ± 0.24

^a^CB-839 and BPTES were used as positive control.

^b^N/A represents not available

^c^n.b. is no clear binding detected; u.p. represents un-performable in the MST measurement due to poor solubility of the compound.

### *In vitro* enzyme inhibition assay

**Figure 3:**
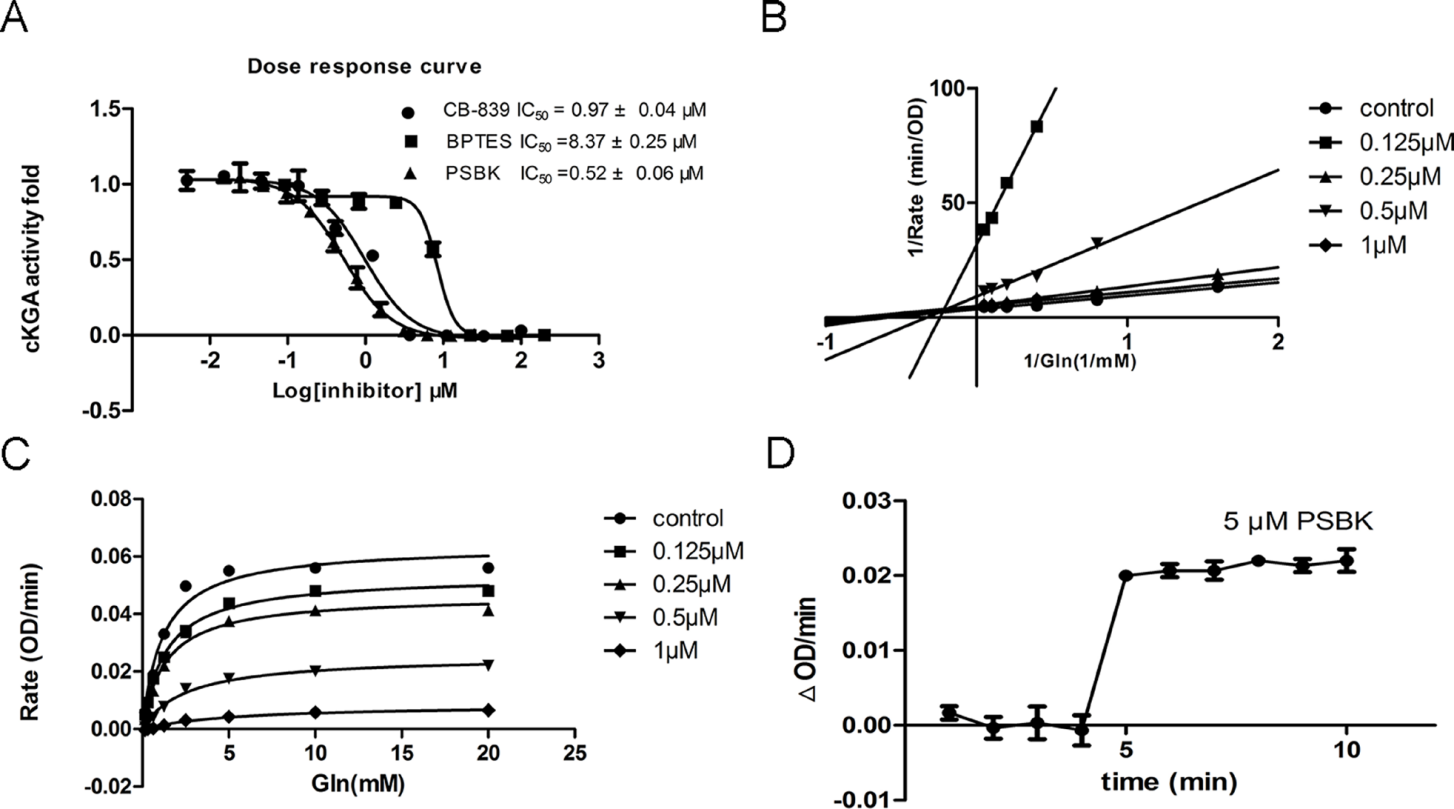
*In vitro* enzyme inhibition assay (**A**) Dose response inhibition of cKGA activity by physapubescin K (PSBK), BPTES and CB-839 were used as positive control. (**B**) Line weaver–Burk double-reciprocal representation of the glutamine saturation profiles for KGA at a range of concentrations of physapubescin K. (**C**) Glutamine saturation profiles for KGA at a range of concentrations of physapubescin K. (**D**) Saturated inhibition assay. The glutamine was 10 mM at 1–4 min and increased to 40 mM at 4 min. Physapubescin K was 5 μM.

To validate the finding of the virtual ligand screening, the catalytic domain of human KGA (cKGA) was expressed by recombinant technique. The enzyme inhibition experiments demonstrated that physapubescin K could significantly inhibit the activity of KGA *in vitro* with IC_50_ value of 0.52 ± 0.06 μM. This was even more potent than the known inhibitors CB-839 and BPTES with IC_50_ of 0.97 ± 0.04 μM and 8.37 ± 0.24 μM, respectively (Figure 3A and Table 2). Enzyme kinetics has been measured to explore the mode of inhibition. As shown in Figure 3B and 3C, physapubescin K displayed a primarily non-competitive inhibition mechanism in low concentration characterized by dose-dependent decrease in Vmax and little effects on Km for glutamine, but showed competitive inhibition in higher concentration characterized by dose-dependent increases in Km for glutamine. As shown in Figure 3D, 5 μM physapubescin K could completely inhibit the activity of KGA with the substrate concentration of 10 mM, however, the catalytic rate of cKGA was immediately increased from 0 to 0.02 ∆OD/min when the substrate concentration was increased from 10 mM to 40 mM. This suggested that the inhibition caused by physapubescin K was reversible.

### Physapubescin K not only binding with recombinant cKGA but also with intracellular KGA

**Figure 4:**
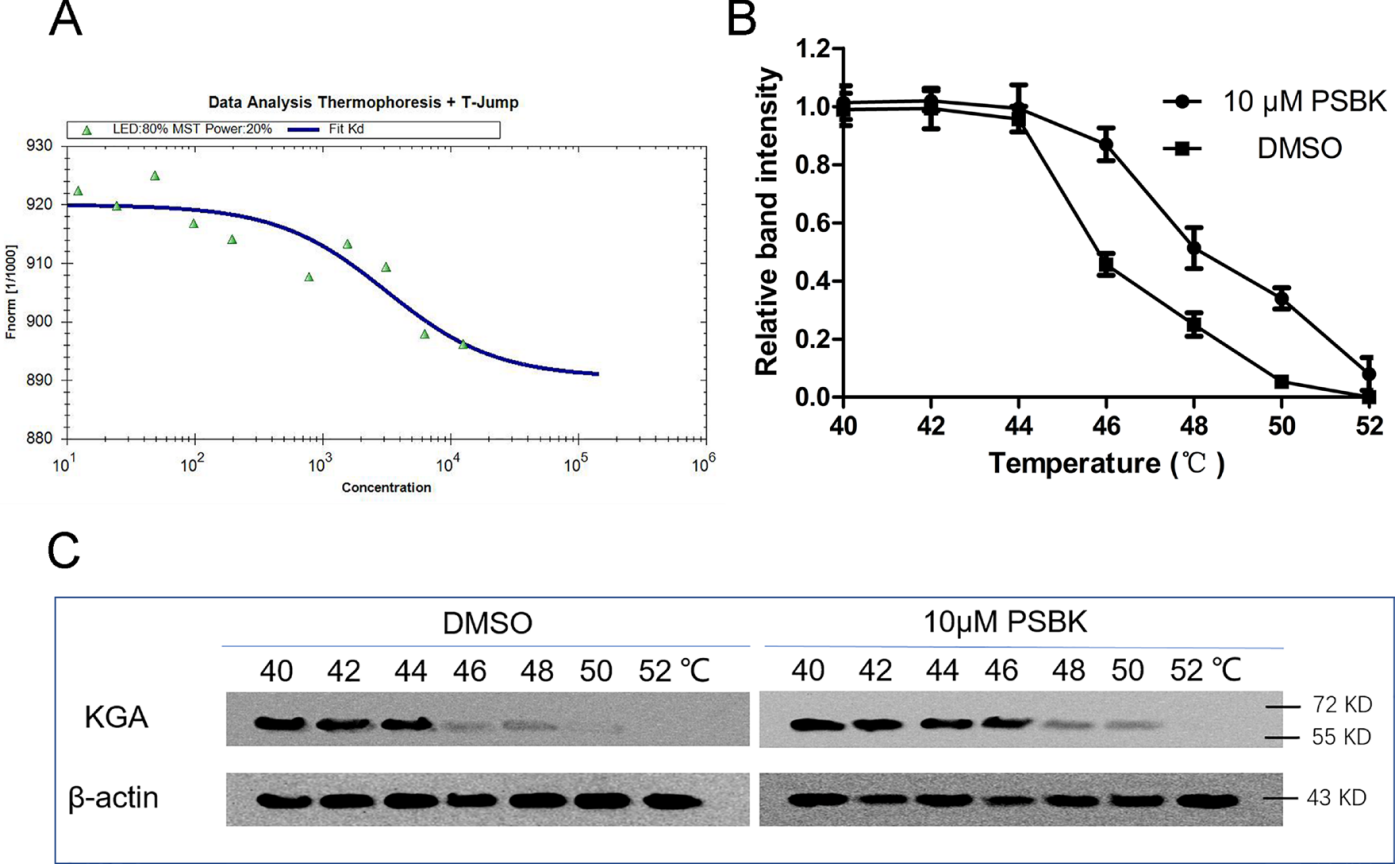
Physapubescin K (PSBK) not only binding with recombinant cKGA but also with intracellular KGA (**A**) Measurement of affinity physapubescin K with KGA by MST in standard treated capillaries, and the resulting binding curve was shown (Kd 3.03 ± 0.38 μM). (**B**–**C**) CETSA was performed on SW1990 cells as described in the Materials and Methods section. The stabilizing effects of physapubescin K on KGA and β-actin at different temperatures were evaluated by Western blot analysis (C). The intensity of the KGA bands was quantified using the Quantity One software (B).

To further investigate the interaction of the compound with KGA, microscale thermophoresis method (MST) was used to assay the binding affinity between physapubescin K and KGA. This technology can quantify protein-protein or protein-small molecule interactions with high sensitivity through detecting fluorescent changes of molecules during thermophoresis [24]. The equilibrium dissociation constant (Kd) of physapubescin K was 3.03 ± 0.38 μM (Figure 4A and Table 2), exhibiting strong binding affinity with cKGA under the buffer condition. However, it was of vital importance to verify whether it could bind with the intracellular KGA. The cellular thermal shift assay (CETSA), which based on the biophysical principle of ligand-induced thermal stabilization of target protein [25], was thus conducted to investigate its affinity with intracellular KGA. The cellular thermal shift assay showed that physapubescin K was able to penetrate cells and stabilize KGA in intact SW1990 cells, indicating its strong affinity with the intracellular KGA. As shown in Figure 4B and 4C, physapubescin K increased the thermal stability of KGA in intact cells from tolerate temperature of 48°C to temperature of 50°C.

### Physapubescin K selectively inhibited the proliferation of glutamine-dependent cells SW1990 and HCC827-ER

**Figure 5:**
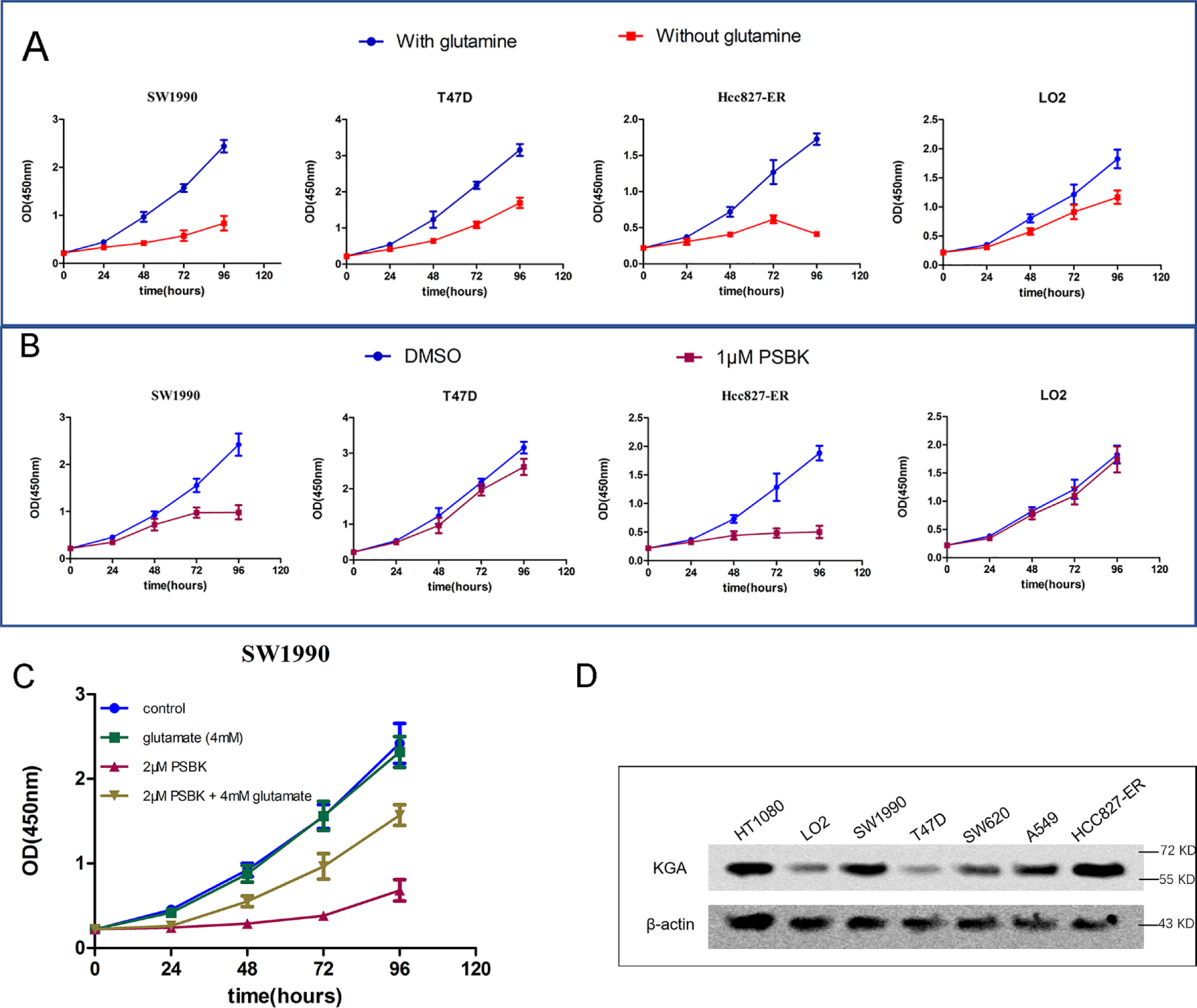
Physapubescin K selectively inhibit the proliferation of glutamine-dependent cells SW1990 and HCC827-ER (**A**) Effect of glutamine deprivation on the proliferation of cancer cells was incubated in DMEM containing 25 mM glucose and 10% FBS, with or without 4 mM glutamine. Data are shown as mean ± SD (*n* = 3 per time point). (**B**) Cells treated with 1 μM PSBK in 0.03% DMSO or with 0.03% DMSO (control). Data are shown as mean ± SD (*n* = 3 per time point). (**C**) SW1990 cells were seeded at 20000 cells/mL with or without PSBK (2 μM) and glutamate (4 mM); viable cells were measured by CCK8 on 24, 48, 36 and 48h, respectively. (**D**) KGA expression levels in various cancer cells.

Effects of glutamine on the growth of four cells were analyzed, including pancreatic ductal carcinoma cell SW1990, erlotinib resistant non-small cell lung cancer cell HCC827-ER, breast cancer cell T47D, human normal liver cell LO2 (Figure 5A). As shown in Figure 5A, compared to that of T47D and LO2 cells, growths of SW1990 and HCC827-ER in glutamine-free media were markedly inhibited, indicating that both cells were glutamine-dependent. In addition, SW1990 and HCC827-ER cells were sensitive to the treatment with 1 μM physapubescin K, but LO2 and T47D cells were not (Figure 5B). Glutamate can be used as the supplementary to compensate for the glutamate deprivation caused by the inhibition of KGA. In order to investigate whether the proliferation inhibitory effects of physapubescin K were mediated by inhibiting glutamine metabolism, glutamate supplementing assay was conducted. As shown in Figure 5C, addition of 2 μM physapubescin K significantly inhibited cell proliferation while addition of 4 mM glutamate did not affect cell proliferation, however, addition of 4 mM glutamate remarkably alleviated the inhibition of cell growth induced by physapubescin K. Moreover, Western blot results showed the over-expression of KGA both in SW1990 and HCC27-ER cells (Figure 5D).

### Physapubescin K inhibited SW1990 cell proliferation both *in vitro* and *in vivo*

**Figure 6:**
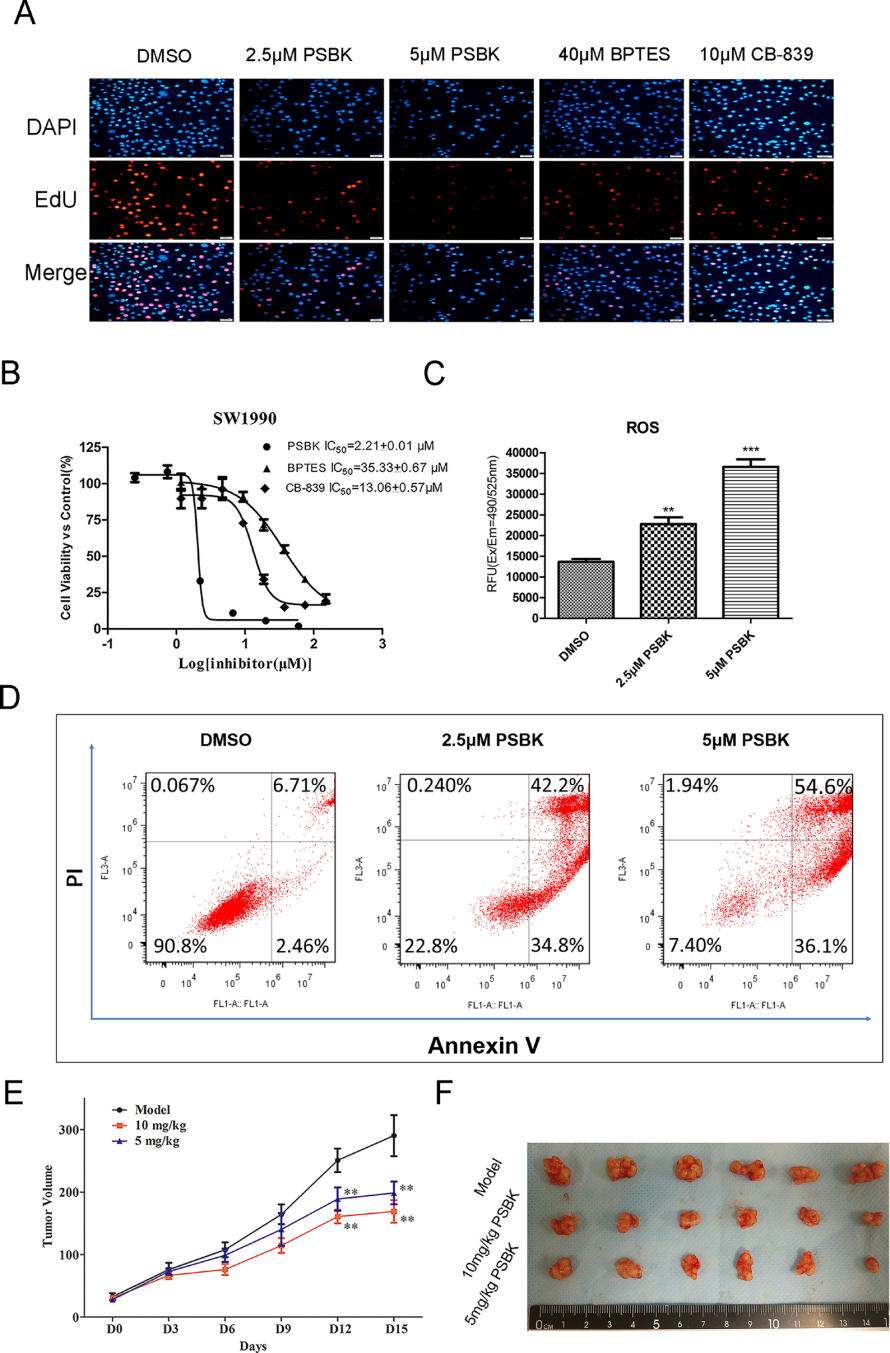
Physapubescin K (PSBK) inhibits SW1990 cell proliferation both *in vitro* and *in vivo* (**A**) DAPI and EdU double staining was used to determine the effect of different concentrations of physapubescin K on the proliferation of SW1990 cells. BPTES and CB-839 were used as positive control. (**B**) The IC_50_ of PSBK to SW1990 measured by CCK8 method was 2.21 ± 0.01 μM. (**C**) SW1990 cell treated with 2.5 μM or 5 μM PSBK in 0.1% DMSO or with 0.1% DMSO (control) for 24 h, The ROS level were measured by fluorescence detection in Ex/Em 490/525 nm. (**D**). Cell apoptotic death analyzed by Annexin V/PI staining. (**E**) The average tumor volumes of vehicle-treated control mice (*n* = 6) and PSBK treated mice (*n* = 6, 5 or 10 mg/kg per day via intraperitoneal injection) were plotted over 15 days after tumor cell injection. (**E**) After 2-week treatment, differences in tumor size are shown. The asterisk ** indicates a significant increased tumor size (*P* < 0.01) in the vehicle-treated group compared with the PSBK-treated group as determined by one-way analysis of variance.

EdU and DAPI double staining experiments showed that physapubescin K significantly inhibited the proliferation of SW1990 cells at the concentration of 5 μM, with the proliferated cell ratio close to zero, which was significantly better than the inhibitory effects of BPTES at 40 μM and CB-839 at 10 μM (Figure 6A). The inhibitory effects of physapubescin K against human normal liver cell LO2, erlotinib-resistant human non-small cell lung cancer cells (HCC827-ER), human pancreatic cancer cells (SW1990), and human fibrosarcoma cells (HT1080) were further investigated using CCK8 method (Table 3). In addition, as shown in Figure 6B, the IC_50_ of this compound against SW1990 was 2.21 ± 0.01 μM, more potential than known inhibitors CB-839 and BPTES with IC_50_ of 13.06 ± 0.57 μM and 35.33 ± 0.67 μM, respectively. What’s more, physapubescin K could significantly increase intracellular reactive oxygen species (ROS) levels and induce apoptosis in SW1990 cells (Figure 6C and 6D).

**Table 3:** IC_50_ values *a* of physapubescin K against human cancer cell lines

No.	Human cancer cell lines^a^			
	LO2	SW1990	HCC827-ER	HT1080
physapubescin K	11.7 ± 0.32	2.21 ± 0.01	0.72 ± 0.22	2.85 ± 0.60

^*a*^Results are expressed as IC_50_ values in μM.

*In vivo* SW1990 xenograft mouse model showed that intraperitoneal injection of 5 mg/kg physapubescin K could significantly inhibit the growth of the tumor (*P* < 0.01) and showed dose-dependent inhibition (Figure 6E and 6F).

### Physapubescin K could block glutamine metabolism

**Figure 7:**
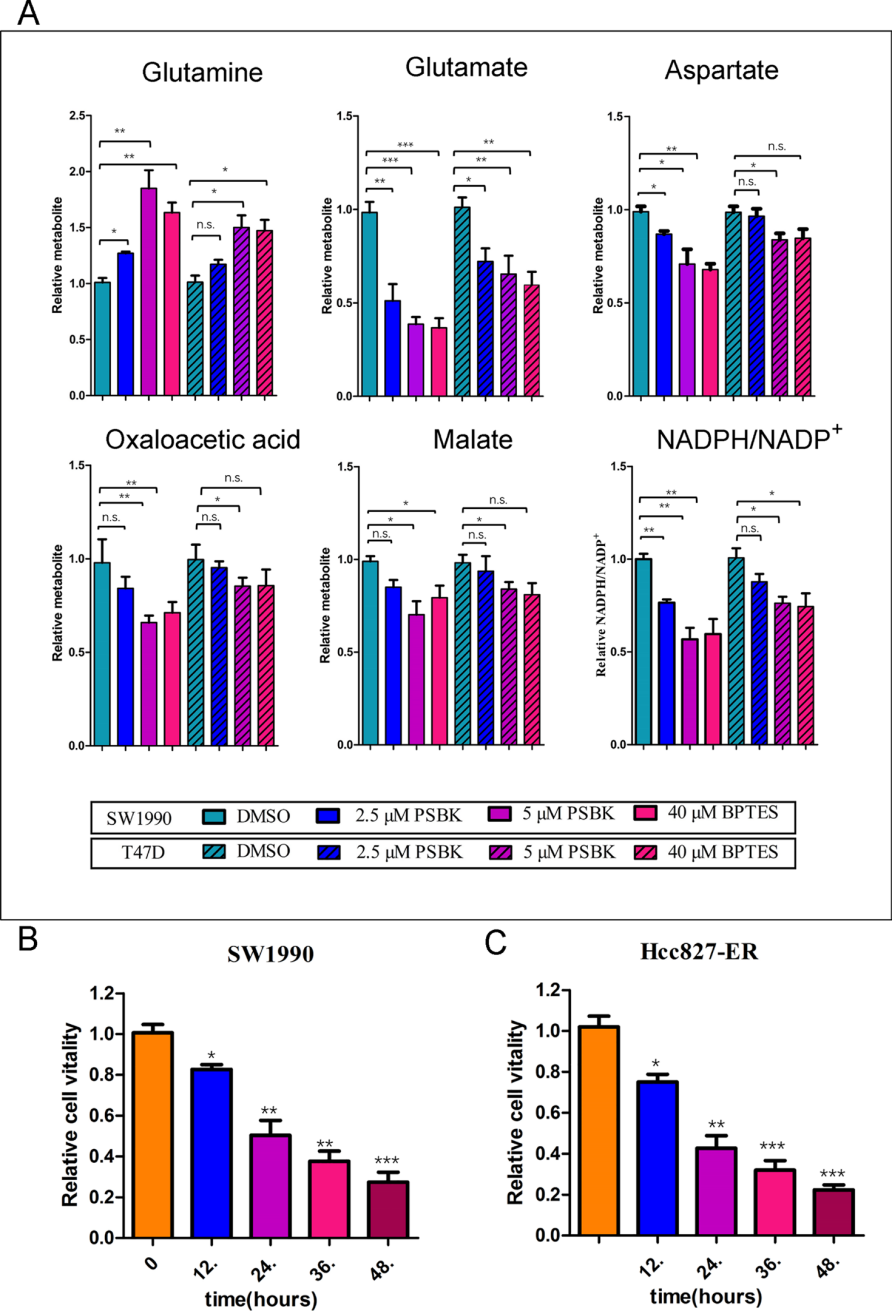
Physapubescin K (PSBK) could block glutamine metabolism (**A**) Relative intracellular metabolite levels and NADPH/NADP^+^ were measured in SW1990 cells or T47D treated with DMSO, 2.5 or 5 μM PSBK for 4 hours. BPTES was used as a positive control. Comparisons of treated and untreated conditions were performed by unpaired *t*-test: *P < 0.05; **P < 0.01; ***P < 0.001; ns means no significant difference. (**B**) After SW1990 cells treated with 2 μM PSBK in 0.06% DMSO or with 0.06% DMSO (control), viable cells were measured by CCK8 on 12, 24, 36 and 48 h. (**C**) After HCC827-ER cells treated with 0.5 μM PSBK in 0.015% DMSO or with 0.015% DMSO (control), viable cells were measured by CCK8 on 12, 24, 36 and 48 h.

Blocking glutamine metabolism can inhibit the growth and proliferation of glutamine-dependent cells, mainly because of blocking the production and utilization of glutamine metabolites. The effects of different concentrations of physapubescin K on the intracellular glutamine and its downstream metabolites were examined. The results showed that it could significantly reduce the consumption of glutamine in the SW1990 cells and decrease the production of glutamic acid and its downstream metabolites including oxaloacetic acid, aspartic acid, and malic acid (Figure 7A). Likewise, physapubescin K significantly reduced the proportion of NADPH/NADP^+^ in SW1990 cells. Whereas, physapubescin K showed moderate effects on glutamine metabolism in T47D cells. These results indicated that physapubescin K blocked glutamine metabolism, thereby reducing downstream products of the tricarboxylic acid cycle involved in providing energy and participating in the synthesis of bio-macromolecules , consequently inhibiting cell growth and proliferation. As shown in Figure 7B, relative cell vitalities of SW1990 treated with PSBK for 12 h, 24 h, 36 h and 48 h groups were 82.6.3%, 50.3%, 37.6%, 27.3%, respectively (^*^P < 0.05; ^**^P < 0.01; ^***^P < 0.001), indicating that the anti-proliferation effect of physapubescin K was in a time dependent manner. And the same effect could also be seen in Hcc827-ER cells (Figure 7C).

### Synergy of physapubescin K in combination treatments

**Figure 8:**
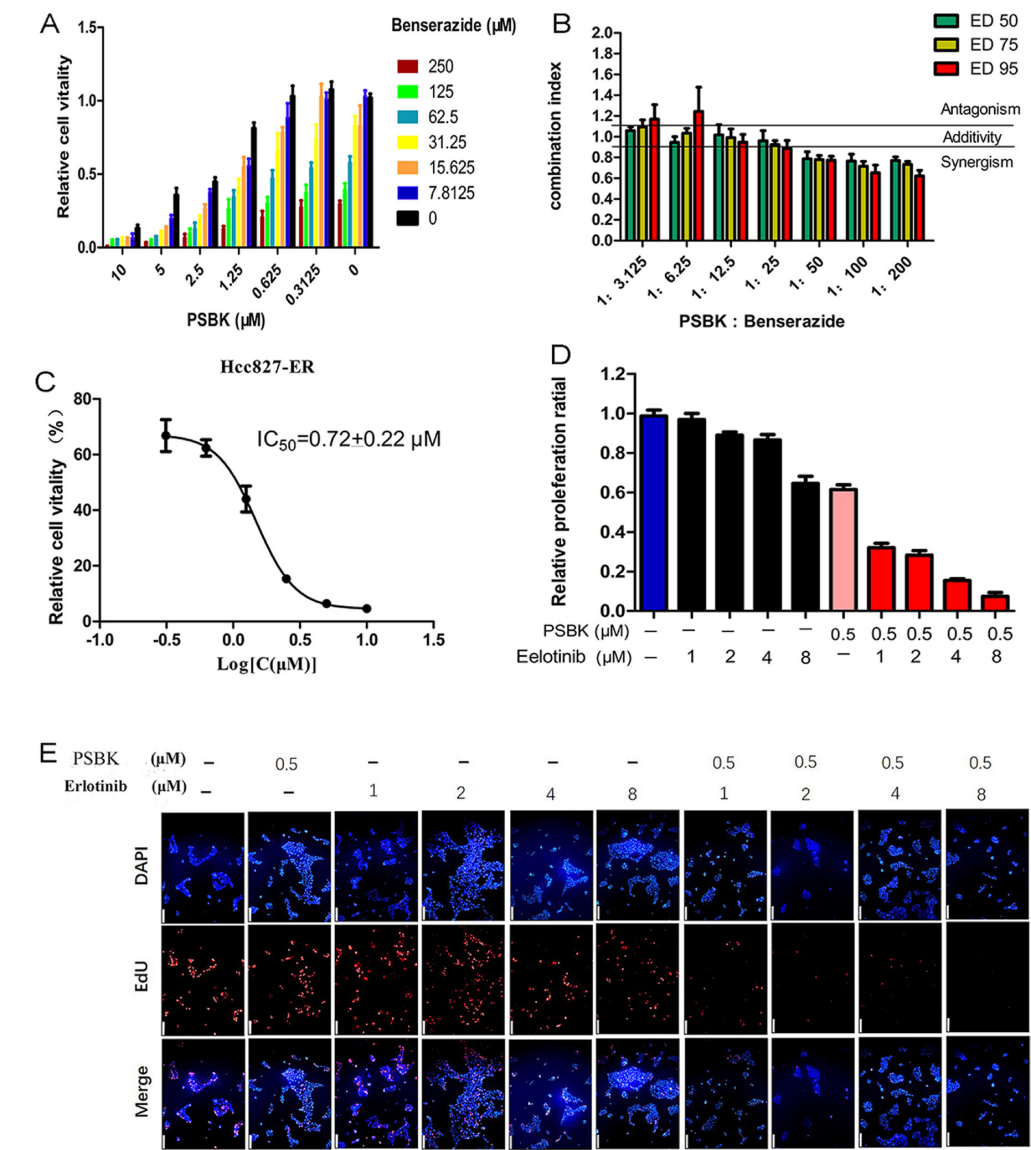
Synergy of physapubescin K (PSBK) in combination treatments (**A**) Cells were cultured for 1 day with or without the indicated doses of PSBK and benserazide, and cell viability was evaluated by CCK8 assay. (**B**) At the indicated concentration ratio of PSBK: benserazide combinations at different effect levels, ED_50_, ED_75_ and ED_95_ were calculated using the CompuSyn software. Bars represent the mean ± SD of two different experiments performed in triplicates. Dashed horizontal lines indicate antagonism (CI > 1.1), additivity (0.9 < CI < 1.1) and synergy (CI < 0.9). (**C**) The IC_50_ of PSBK to HCC827-ER measured by CCK8 method was 0.72 ± 0.22 μM. (**D**, **E**) DAPI and EdU double staining was used to determine the effect of indicated doses of PSBK and erlotinib on the proliferation of SW1990 cells (E). The proportion of cells in proliferation is counted by manual counting (D).

Tumor cells are speculated to maintain growth and proliferation through a variety of energy metabolism pathways. Therefore, targeting at a single energy metabolism material might not be an effective method for cancer therapy. It has been determined that growing cancer cells were strongly dependent on energy sources such as glucose and glutamine [26]. We supposed that the blocking of both glucose and glutamine metabolism pathways could display a synergistic tumor inhibition. Hexokinase 2 (HK2) was the crucial enzyme controlling the first step of glycolysis, and systemic deletion of HK2 can impair the tumor progression in mouse models. Nowadays, Metformin (Met), 2-Deoxyglucose (2-DG) and 3-Bromopyruvate (3-BrPA) are most commonly reported HK2 inhibitors. Because of the poor potency and side-effects of these compounds, developing novel potent HK2 inhibitors is a matter of great urgency. Benserazide was unexpectedly characterized as an HK2 inhibitor in our previous study [27]. The combined inhibitory effects of physapubescin K and benserazide on the growth of SW1990 cells were evaluated. As shown in Figure 8A, their combination could significantly enhance growth inhibitory activities against SW1990 cells. Figure 8B showed that combined use of physapubescin K and benserazide with ratio less than 1:50 to produce synergistic effects with a CI value less than 0.8. The synergistic mechanism needs to be further investigated and the synergistic effect should be verified by *in vivo* models in future studies.

The epidermal growth factor receptor (EGFR) tyrosine kinase inhibitor (TKI) erlotinib has been used to treat non-small cell lung cancer (NSCLC) in clinic for a decade. However, cancer cells become resistant to the drug through various mechanisms. It was reported that erlotinib has an inhibitory activity against non-small cell lung cancer cell line HCC827 with IC_50_ of 1 nM. Whereas HCC827-ER cell line could tolerate micromole level of erlotinib [28–30]. Previous study found that combination of GLS1 inhibitors and erlotinib markedly reduced EGFR expression despite weak to no effects of erlotinib or GLS1 inhibitor alone on EGFR expression. Thus, blocking glutamine metabolism could improve the sensitivity of HCC827-ER to erlotinib [28].

In this study, the inhibitory effects of different concentrations of erlotinib (1, 2, 4, 8 μM) with physapubescin K (0.5 μM) on cell proliferation were compared with physapubescin K alone and erlotinib alone by double staining of EdU and DAPI. The results showed that physapubescin K could significantly increase the sensitivity of HCC827-ER to erlotinib (Figure 8D and 8E). The dose-dependent inhibition curve of physapubescin K on HCC827-ER was shown in Figure 8C.

## DISCUSSION

It was reported that the growth of cancer cells was strongly dependent on energy sources such as glucose and glutamine [31]. Kidney-type glutaminase (KGA), a mitochondrial enzyme, was over expressed in many cancers, which has been determined as a promising target for cancer therapy in recent years [6]. To date, intense efforts have been made to develop the specific KGA inhibitors, which resulted in synthesis of DON, BPTES and CB-839 [7, 14]. Since de novo designing new targeted antitumor drug was not an easy task, natural products and their derivatives were another alternative way to obtain active lead compounds. Structure-based virtual ligand screening facilitated discovery of small-molecule KGA inhibitors in early stage. As our ongoing studies on natural KGA inhibitors [19], a new withanolide-type inhibitor physapubescin K was found from a small in-house database of natural products through the above method. *In vitro* enzyme inhibition assay displayed that physapubescin K inhibited KGA in nanomole level that validated the results of virtual screening. MST assays quantitatively measured the affinity between physapubescin K and recombinant KGA protein, and cellular thermal shift assay (CETSA) further confirmed its strong affinity with the intracellular KGA. CCK8 assay displayed that physapubescin K could selectively inhibit glutamine dependent cancer cell proliferation with dose and time dependent manner. According to previous studies, blocking glutamine metabolism can induce apoptosis under oxidative stress. Analogues of physapubescins K, like physapubescins and physapubescins B [32, 33], have been reported to possess the effect of promoting cell apoptosis. Similarly, our study demonstrated that physapubescins K could also induce apoptosis with significantly increased ROS levels. Combined with our previous study [19], the 4β-hydroxy-2-en-1-one group in ring A and 5β,6β-epoxy group in ring B, were essential for their antitumor effects, which was in agreement with previous reports on structure-activity relationship [21, 34].

It is worthy of mentioning that physapubescin K showed anti-proliferative activity in a PDCA cell line SW1990 both *in vitro* and *in vivo*. It blocked SW1990 cells in glutamine consumption by increasing intracellular level of glutamine and decreasing glutamate and its downstream metabolites. Further assay demonstrated that this inhibition of cell proliferation could be reversed by the addition of glutamate.

A combination of therapies to treat malignancies was at the forefront of research with the aim to reduce drug dosage and side effects, as well as diminish the possibility of resistance emergence. Aerobic glycolysis was an important cancer metabolism pathway, in which hexokinase 2 (HK2) was a crucial enzyme controlling the first step of glycolysis. Benserazide was identified as a novel HK2 inhibitor in our previous study [27]. In this investigation, physapubescin K showed synergistic inhibitory effects in combination with benserazide on SW1990 cells, suggesting that targeting at both glucose and glutamine metabolism pathways could produce improved efficacy and cytotoxicity with smaller dose of both compounds. More detailed synergistic mechanisms of physapubescin K and benserazide still need to be further investigated.

Interestingly, physapubescin K exhibited potent inhibitory effects on the erlotinib-resistant human non-small cell lung cancer cell line HCC827-ER with an IC_50_ of nanomolar grade. What is more, physapubescin K could reverse the resistance of HCC827-ER cells to erlotinib, showing a synergistic effect. In this regard, physapubescin K may be further developed as a potent sensitizer for HCC827-ER cells to erlotinib, which could be used in combination with erlotinib for the treatment of erlotinib-resistant non-small cell lung cancer. All these tend to imply that physapubescin K is at least a good lead compound for designing novel KGA inhibitors. As far as we know, physapubescin K, a new withanolide-type compound is the first kind of natural KGA inhibitor, which was found in our ongoing phytochemical investigations on *P. pubescens* L. In our future study, more pre-clinical evaluations will be further carried out.

## MATERIALS AND METHODS

### Extraction and isolation

The dried stem and leaves of *P. pubescens* L. (50 Kg) collected from Shenyang, Liaoning Province, China, were cut into small pieces and extracted with 75% ethanol (500 L × 2). The resulting extract (6.5 kg) was concentrated in vacuo, suspended in H_2_O (12 L), and partitioned successively with petroleum ether (12 L × 3), EtOAc (12 L × 3), and *n*-BuOH (12 L × 3) to give three fractions. The EtOAc fraction (537 g) was subjected to a silica gel CC (14 × 100 cm) that was eluted with CH_2_Cl_2_/CH_3_OH (100:1, 40:1, 20:1, 10:1, 4:1, 2:1, 1:1 and 0:1 v/v) to obtain nine combined subfractions (E1−E9). Fraction E3 (38 g) was subjected to a silica gel column (8 × 50 cm) and eluted with CH_2_Cl_2_/acetone (from 100:1 to 1:1, v/v) to produce eight subfractions (E31−E38). E36 (7.8 g) was chromatographed repeatedly on a Sephadex LH-20 column (3.0 × 80 cm) that was eluted with CH_2_Cl_2_/CH_3_OH (1:1, v/v) and further purified via preparative HPLC (60% CH_3_OH/H_2_O) to yield physapubescin K (750 mg).

### Molecular docking

Crystal structure of human KGA (PDB code: 3VP1) was obtained from the Protein Data Bank. The docking was operated by ICM 3.8.2 modeling software on an Intel i7 4960 processor (MolSoft LLC, San Diego, CA). And ligand binding pocket residues were selected by graphical tools in ICM software to create the boundaries of docking search. The glucose was the co-crystallized ligand in this crystal structure for docking, and it was deleted when setting up the receptor. In docking calculation, potential energy maps of the receptor were calculated using default parameters. Compounds were inputted into ICM and an index file was created. Conformational sampling was based on the Monte Carlo procedure 30, and finally the lowest-energy and the most favorable orientation of the ligand was selected [23, 35].

### Cloning, expression and purification of KGA

Specific details were referenced to our previous work [19]. Briefly, The catalytical domain of human KGA (cKGA) encoding amino acids 221–553 was cloned into pET26b vector with C-terminal His tag. The cKGA was then expressed in *E.coli* BL21 (DE3)-RIL-Condon plus. Cultures were grown in LB media contain 35 mg/L kanamycin at 37°C to OD value in 0.6–0.8, then induced at 16°C with 0.4 mM IPTG for 16 h. Cultures were harvested by centrifugation at 3500 g for 8 minutes. Cell pellets were sonicated in a buffer [6] containing 50 mM Hepes (pH 7.5), 500 mM NaCl, 5mM imidazole, 10 glycerol, 1 mM DTT, 0.1% Triton X-100, and final 1 mM PMSF (Sigma-Aldrich USA). The cKGA were purified by affinity chromatography by Ni-NTA agarose, and further purified by size exclusion chromatography (Superdex 200, GE-health care, USA). The purified cKGA was stored in buffer containing 20 mM Hepes (pH 7.5), 200 mM NaCl, 10% glycerol and 3 mM DTT.

### *In vitro* IC_50_ assays for KGA inhibition

The KGA profiling assays were to evaluate the compound-dependent inhibition of KGA *in vitro* catalytic assay [17]. Specific details were referenced to our previous work [19]. Briefly, the KGA catalyzed the glutamine into glutamate plus ammonium, and then glutamate plus NAD converted to α-ketoglutarate and NADH by the catalysis of GDH. The elevated absorbance at 340 nm with the form of NADH was recorded using a microplate reader. The enzymatic activity was measured in 100 μL system. Mixed buffer containing 0.15 M K_2_HPO_4_, 50 mM Tris-Acetate pH 8.6, 0.1 mg/mL bovine serum albumin (BSA), 0.25 mM EDTA, 1 mM DTT, 4 mM NAD were prepared. Then, 10 mM glutamine, 1 unit of glutamate dehydrogenase (GDH) and inhibitor were added in the mix. Final concentration of 1 μM cKGA was added to initiate the reaction. Generation of NADH was monitored by 340 nm absorbance every minute for 20 minutes at 37°C. The IC_50_ values were calculated by fitted regression equation using the-log plot (GraphPad Prism). Each value was on behalf of the means ± SD of three independent tests, each with three replicates.

### Microscale thermophoresis (MST) Assay

The ability of the compound to bind with purified KGA was analyzed with MST. Specific details were referenced to our previous work [19]. Briefly, The protein was labeled with the Monolith NT™ Protein Labeling Kit RED (Cat#L001) according to the manufacturer’s instructions. Labeled KGA were diluted in a 20 mM HEPES (pH 7.5) and 0.05 (v/v)% Tween-20 at 200 nM. Compounds were diluted in a range of concentration steps and incubated with KGA for 10 min at room temperature. Samples were loaded into Monolith TM standard-treated capillaries and the thermophoresis was carried out at 26°C after 10 min incubation on a Monolith NT.115 instrument (Nano Temper Technologies, München, Germany). Data were obtained with 100% LED power and 20% MST. Kd values were fitted by using the NT Analysis software (Nano Temper Technologies, München, Germany).

### Cellular thermal shift assay (CETSA)

The ability of compounds to interact with, and thereby stabilize the target in intact cells, was analyzed essentially as described by Alshareef A *et al.* [36]. Briefly, cells cultured in 10-cm tissue culture dishes at about 90% confluence were treated with media containing DMSO or 10 μM physapubescin K for 4 hours. After treatment, cells were detached with cell scraper, collected by centrifugation and subsequently resuspended in PBS. The cell suspension was aliquoted into seven PCR tubes and heated for 3 minutes to 40, 42, 44, 46, 48, 50 or 52°C. Subsequently, cells were lysed using liquid nitrogen and two repeated cycles of freeze-thaw. Precipitated proteins were removed by centrifugation at 13,000 g for 20 minutes. Soluble proteins, collected in the supernatant, for further Western blot analysis.

### Cytotoxicity assay

The HCC827-ER cell line was kindly provided by Professor Jianbin Wang (Institute of Translational Medicine, Nanchang University, China). The SW1990, HT1080, and A375-S2 human tumor cell lines were obtained from ATCC (Manassas, VA, USA). HCC827-ER cells were cultured in RPMI-1640 medium with 10% fetal bovine serum (FBS). SW1990, HT1080, and LO2 cells were cultured in DMEM medium with 10% fetal bovine serum (FBS). All of these cells were cultured at 37°C in a humidified atmosphere with 5% CO_2_. To estimate cell viability, cells (5000/well) were separately seeded in 96-well plates for 24 h at 37°C in a 5% CO_2_ incubator. The attached cells were fed with fresh medium containing various concentrations of physapubescin K (0–100 μM) for additional 48 h, using BPTES as the positive control. After culturing for various times, the cytotoxicity of physapubescin K was measured using a CCK8 assay kit according to the manufacturer’s instructions. All the experiments were performed in triplicate, and the mean absorbance values were calculated. The IC_50_ values of the compounds were derived from the mean OD values of the triplicate tests versus the drug concentration curves.

### EdU and DAPI double staining to detect cell proliferation

The logarithmic growth phase cells were seeded in 24-well plates at 2 × 10^4^ ∼ 5 × 10^5^ cells per well. After 4–6 h, the medium was removed, the medium containing no or different concentrations of compound was replaced, and 50 μM of EdU was added. 2 h later, the medium was removed, cells washed by PBS. 200 μL of cell fixative (i.e., PBS containing 4% paraformaldehyde) was added and incubated at room temperature for 30 minutes. The solution was discarded and 200 μL of 2 mg/mL glycine was added to each well. After 5 minutes of incubation, the glycine solution was discarded. Each well washed with 200 μL of PBS, rinse for 5 minutes, and PBS was removed. Apollo staining was added to each well with 200 μL of 1 X Apollo^®^ staining solution. After incubation for 30 minutes at room temperature and decolorization, the reaction solution was discarded. Similarly, 200 μL of 1 × Hoechst 33342 solution was added to each well to dye the total DNA. After 30 minutes of incubation at room temperature and the reaction solution was discarded, and then the images were captured and analyzed by fluorescence microscopy.

### Cell proliferation assay

SW1990, HCC827-ER, T47D and LO2 cells were seeded at a density of 2 × 10^3^ per well into 96-well plates. Cell proliferation was assessed by incubating them with Cell Counting Kit-8 reagents (Dojindo, Shanghai, China) for 24 h, 48 h, 72 h, 96 h at 37°C and measuring the absorbance at 450 nm. The experiment was performed in triplicate.

### Western blot analysis

The harvested cells were lysed using liquid nitrogen and two repeated cycles of freeze-thaw. Insoluble debris was removed by centrifugation and the concentration of total proteins was determined using BCA Protein Assay Kit (Beyotime, China). Then lysate protein (20–40 μg) was subjected to 10% SDS-PAGE and electrophoretically transferred to polyvinylidene difluoride membranes (PVDF) (Millipore, USA). The membranes were sequentially blocked with 5% non-fat milk and incubated overnight with the following primary antibodies: β-actin (A2228), KGA (20170-1-AP). Protein bands were visualized using an enhanced chemiluminescence reagent (ECL Plus) (GE Healthcare, USA) after hybridization with a HRP conjugated secondary antibody. All experiments were performed in triplicate and analyzed by Quantity One software.

### Measurement of intracellular metabolism level

NADPH/NADP^+^ ratio was determined by using NADP^+^/NADPH Quantification Colorimetric Kit (BioVision, #K347-100). The abundance of intracellular glutamine (KeyGen, Nanjing, China A124), glutamate (KeyGen, Nanjing, China A074), (BioVision, #K778), aspartate (BioVision, #K552), oxaloacetate (BioVision, #K659) and malate (BioVision, #K637) was determined by using quantification kits, according the manufacturer’s instruction. Briefly, 5 × 10^6^ cells (10-cm dishes) were collected during log-phase growth by cell scraper, homogenized in 0.2 mL of provided buffers (on ice), and centrifuged at 4°C for 10 min at 13,000 g. Supernatants were deproteinized using 10 K Ultrafiltration tube (Millipore, USA), analyzed and compared to standard curves. The signals obtained were normalized to the protein concentration calculated upon processing a parallel 10-cm dish.

### ROS Quantification

DCFDA assay was performed 24 h after interfered with difference concentration of physapubescin K or DMSO. Cells were incubated with 5 μM 2′,7′-dichlorodihydrofluorescein diacetate (DCFDA, KeyGen, Nanjing, China A124) for 30 min. Cells were washed twice with PBS to remove excess DCFDA, and the labeled cells were then collected in PBS. The fluorescent of 2′,7′-dichloro-fluorescein (DCF) was proportionate to ROS generation and was measured by microplate reader.

### Flow cytometric analysis for apoptosis

SW1990 cells were cultured in six-well plates and treated with different concentrations of physapubescin K for 24 h. Then the cells were harvested, washed twice with ice-cold PBS, and mixed in 100 μL of 1× binding buffer (10 mM HEPES/ NaOH, pH 7.4, 140 mM NaCl, 2.5 mM CaCl_2_). After incubating with Annexin-V and PI staining liquid (Nanjing KeyGen Biotech. Inc.) for 15 min at room temperature, the cells were examined by flow cytometry (BD Biosciences, FACSCalibur).

### Antitumor efficacy of physapubescin K in xenograft mouse model *in vivo*

All animal experiments were performed in accordance with the Guide for the Care and Use of Laboratory Animals of Tongji Medical College, Huazhong University of Science and Technology and approved by the Ethics Committee. CB-17/ SCID mice (male, 4 weeks old) were purchased from Beijing HFK Bioscience CO., LTD (Beijing, China). SW1990 cells were inoculated subcutaneously (3 × 10^6^ cells) into the left flank of each mouse. Six days later, mice were randomly divided into six groups and were injected i.p. daily for 16 days with one of the following treatments: (1) natural saline group (*n* = 10); (2) 5 mg/Kg physapubescin K (*n* = 10); (3) 10 mg/Kg physapubescin K (*n* = 10). The dose volume was 0.1 mL/10 g body weight, and the weights of mice were recorded every day. Meanwhile, the tumor volumes were measured with vernier calipers and calculated by the following formula: (A × B2)/2, where A was length and B was width of the two-dimension tumor. After animals were sacrificed, tumor weights were measured. The inhibition ratio (%) was calculated using the following equation: I% = 100% × [W_tumor (vehicle)_ − W_tumor (treated)_]/W_tumor (vehicle)_

### Statistical analysis

Statistical analysis of the data was performed using Graph Pad Prism 5.0 software. The data were expressed as the means ± SD. Values were analyzed using SPSS version 12.0 software by one-way analysis of variance (ANOVA), and *p* < 0.05 was considered statistically significant.

## Abbreviations

KGA: Kidney-type glutaminase
PSBK: physapubescin K
PDAC: pancreatic ductal adenocarcinoma
MST: Microscale thermophoresis
CETSA: The cellular thermal shift assay
CCK-8: Cell Counting Kit-8
PVDF: Polyvinylidene difluoride membranes
